# Inhibition of microglial β-glucocerebrosidase hampers the microglia-mediated antioxidant and protective response in neurons

**DOI:** 10.1186/s12974-021-02272-2

**Published:** 2021-09-22

**Authors:** Electra Brunialti, Alessandro Villa, Marianna Mekhaeil, Federica Mornata, Elisabetta Vegeto, Adriana Maggi, Donato A. Di Monte, Paolo Ciana

**Affiliations:** 1 Department of Health Sciences, University of Milan, Milan, Italy; 2 Department of Pharmaceutical Sciences, University of Milan, Milan, Italy; 3 German Center for Neurodegenerative Diseases (DZNE), Bonn, Germany

**Keywords:** Parkinson’s disease, Gaucher’s disease, microglia, NFE2L2, Neuroprotection

## Abstract

**Background:**

Homozygotic mutations in the GBA gene cause Gaucher’s disease; moreover, both patients and heterozygotic carriers have been associated with 20- to 30-fold increased risk of developing Parkinson’s disease. In homozygosis, these mutations impair the activity of β-glucocerebrosidase, the enzyme encoded by GBA, and generate a lysosomal disorder in macrophages, which changes morphology towards an engorged phenotype, considered the hallmark of Gaucher’s disease. Notwithstanding the key role of macrophages in this disease, most of the effects in the brain have been attributed to the β-glucocerebrosidase deficit in neurons, while a microglial phenotype for these mutations has never been reported.

**Methods:**

We applied the bioluminescence imaging technology, immunohistochemistry and gene expression analysis to investigate the consequences of microglial β-glucocerebrosidase inhibition in the brain of reporter mice, in primary neuron/microglia cocultures and in cell lines. The use of primary cells from reporter mice allowed for the first time, to discriminate in cocultures neuronal from microglial responses consequent to the β-glucocerebrosidase inhibition; results were finally confirmed by pharmacological depletion of microglia from the brain of mice.

**Results:**

Our data demonstrate the existence of a novel neuroprotective mechanism mediated by a direct microglia-to-neuron contact supported by functional actin structures. This cellular contact stimulates the nuclear factor erythroid 2-related factor 2 activity in neurons, a key signal involved in drug detoxification, redox balance, metabolism, autophagy, lysosomal biogenesis, mitochondrial dysfunctions, and neuroinflammation. The central role played by microglia in this neuronal response in vivo was proven by depletion of the lineage in the brain of reporter mice. Pharmacological inhibition of microglial β-glucocerebrosidase was proven to induce morphological changes, to turn on an anti-inflammatory/repairing pathway, and to hinder the microglia ability to activate the nuclear factor erythroid 2-related factor 2 response, thus increasing the neuronal susceptibility to neurotoxins.

**Conclusion:**

This mechanism provides a possible explanation for the increased risk of neurodegeneration observed in carriers of GBA mutations and suggest novel therapeutic strategies designed to revert the microglial phenotype associated with β-glucocerebrosidase inhibition, aimed at resetting the protective microglia-to-neuron communication.

**Supplementary Information:**

The online version contains supplementary material available at 10.1186/s12974-021-02272-2.

## Background

The human GBA gene encodes the lysosomal β-glucocerebrosidase (GCase) enzyme [1]. Approximately 300 pathogenic mutations in the coding regions of GBA have been described, each of which affects GCase activity to a different extent; the effects range from negligible to a severe reduction in activity [2]. The enzymatic deficiency in homozygous individuals leads to the accumulation of glucosylceramide in lysosomes of macrophage/monocyte cells, causing Gaucher’s disease [3]. Notably, GBA mutations represent the most significant genetic risk factor for Parkinson’s disease since carriers display a 20- to 30-fold increased risk (depending on the genetic variant) of developing the illness [4]. Parkinson’s patients with GBA mutations exhibit pathological hallmarks and clinical manifestations comparable to those of idiopathic individuals, supporting the notion that common dysfunctional mechanisms can trigger dopaminergic neurodegeneration in idiopathic and GBA-deficient patients [5]. This interrelation goes beyond GBA mutations since low GCase activity has been found in all studied cases, even in patients without GBA mutations [5, 6]. Several hypotheses have been formulated for the involvement of GCase impairment in Parkinson’s neurodegeneration (for a detailed review see [5]); however, the picture is still incomplete, as the hypotheses do not explain why not all GBA carriers develop Parkinson’s disease [5]. The reported studies have focused mainly on the roles of GBA mutations in neurons. Intriguingly, however, it has been discovered that GBA deficiency around dopaminergic neurons is not sufficient to induce neuronal death and pathological motor behavior in mice [7]. This evidence suggests that GBA deficiency affects the functions of other cells to trigger parkinsonism [7]. In this regard, brain-resident microglia seem to be the perfect candidates: microglia are essential cells in brain homeostasis maintenance, and microglial dysfunction is strictly associated with neurological problems and neurodegeneration [8]. Microglia is the cell lineage in the brain which expresses the highest amount of Gba mRNA [9], and as the phlogistic brain counterparts sharing common functions with macrophages [10], it is conceivable that these cells are affected by GCase impairment, as macrophages are in Gaucher’s disease [3].

In the late pathological phases, when neurological death is evident, microglial activation is a common feature in both Parkinson’s and Gaucher’s disease; indeed, neuroinflammation seems to be an important factor propelling neurodegeneration in Gaucher’s disease [11–14]. In addition, in the postmortem substantiae nigrae of Parkinson’s patients, activated microglia and increased concentrations of proinflammatory cytokines have been identified [15, 16]. Interestingly, increasing GCase levels in the brain via administration of isofagomine results in a reduction in the microglial inflammatory response and an improvement in motor function in transgenic mice overexpressing α-synuclein in the substantia nigra [17].

Although the role of proinflammatory microglia in the late pathological stages is clear, it seems that microglia also play roles in the early pathological phases preceding the death of neurons; accordingly, in a *Danio rerio* Gaucher disease model, it has been found that microglial activation precedes neuronal cell death. More captivatingly, in GBA mutation carriers without prodromal or clinical Parkinson’s manifestation, positron emission tomography (PET) scans have revealed microglial activation in Lewy-susceptible brain regions [18].

GCase inhibition in cellular and animal models increases susceptibility to exogenous or endogenous parkinsonism-inducing stimuli, including 1-methyl-4-phenyl-1,2,3,6-tetrahydropyridine (MPTP) treatment, rotenone treatment, and α-synuclein overexpression [19–25], supporting the hypothesis that defective detoxification and neuroprotective responses could be present in GCase-deficient brains. A well-recognized player in neuronal defense against neurotoxic insults and oxidative stress is the transcription factor nuclear factor erythroid 2-related factor 2 (NFE2L2, also known as NRF2) [26]. This leucine zipper protein is normally kept in the cytoplasm in an inhibitory complex with kelch-like ECH-associated protein 1 (KEAP1) and cullin 3; when stimulated, NFE2L2 is released from the complex and translocates into the nucleus, where it binds to promoters and regulates the transcription of target genes [27]. Notably, NFE2L2 dysregulation has been associated with neurodegenerative processes. Recently, we demonstrated that NFE2L2 is activated early in neurons and glial cells during dopaminergic neurodegeneration, well before the onset of neuronal death [28], suggesting that this neuroprotective mechanism has a pivotal role in the early pathogenic stages. In the present work, we aimed to clarify whether GBA impairment interferes with the activation of this microglia-mediated neuroprotective pathway, thus increasing the susceptibility of the brain to neurotoxic insults. Our results demonstrate the existence of a novel mechanism of neuroprotection based on the physical microglia/neuron interaction that leads to activation of the NFE2L2 pathway in neurons. Inhibition of microglial GCase activity hampers this communication, suggesting that the microglial GBA gene product actively participates in the induction of detoxifying signals in neurons.

## Methods

### Reagents

All of the following reagents were purchased from Merck: tert-butylhydroquinone (tBHQ, Cat. 112941), conduritol-B-epoxide (CBE, Cat. 234599), cytochalasin D (Cat. C8273), and nocodazole (Cat. M1404). PLX3397 was purchased from DBA (Cat. S7818-50).

### Cell cultures

All cell lines were purchased from the American Type Culture Collection (ATCC). Primary neurons were derived from neural tissue of the cerebral cortex from p0-1 mice using a Neural Tissue Dissociation Kit-Postnatal neurons (Cat. 130-094-802, Miltenyi Biotec) following a standard procedure. After removing the meninges, the brain cortices from six mice were pooled as a single experimental group and subjected to enzymatic and mechanical dissociation at 37 °C. The cellular suspensions were filtered with a 70-μm strainer and seeded on poly-L-ornithine-coated plates. Half of the medium volume was replaced every 2 or 3 days. Microglia were isolated from whole brains of adult (age 3–6 months) male mice with a previously described protocol [29]. The brains from two mice were pooled and subjected to enzymatic and mechanical dissociation. After myelin removal, the cells were processed for sorting with a magnetic column to purify CD11b+ cells, namely, microglia (Cat. 130-093-634, Miltenyi Biotec). To generate human blood-derived macrophages, we followed a previously described procedure [30]. Human peripheral blood mononuclear cells were isolated from buffy coats obtained from healthy volunteers (Niguarda Hospital) after Ficoll-Paque® Plus (Cat. 17-1440-02, GE Healthcare) density gradient centrifugation. Before use, the peripheral blood mononuclear cells were grown for 1 week in DMEM (Cat. 32430-027, Gibco) supplemented with 10% fetal bovine serum (FBS) (ultralow endotoxin, ECS0186L, Euroclone), 1% streptomycin–penicillin (Cat. 15240-062, Gibco), 1% GlutaMAX (Cat. 35050-061, Gibco), and 50 ng/mL M-CSF (Cat. 11343113, Immunotools), and the medium was exchanged every 2 days. Primary peritoneal murine macrophages were extracted as described in [31]. Three mice at 3–6 months of age were euthanized and subjected to peritoneal lavage with PBS to extract macrophages, which were subsequently purified using CD11b+ magnetic beads (Cat. 130-049-601, Miltenyi Biotec), following the manufacturer’s instructions. Human macrophages were detached from the plate using StemProAccutase (Cat. A11105, Gibco), centrifuged and resuspended in RPMI complete medium. SK-ARE-*luc2* cells were obtained by stable transfection of SK-N-BE cells. Briefly, cells were transfected with pARE-*luc2*-ires-tdTomato [28] and pSTC1-Neo DNA plasmids (10:1 ratio) using Lipofectamine LTX & PLUS reagent (Cat. 15338, Thermo Fisher Scientific) with a DNA:Lipofectamine LTX:PLUS reagent ratio of 2.5:6.5:1.5 following the manufacturer’s instructions. Forty-eight hours after transfection, cells were reseeded at different concentrations, and positive clones were identified by selection using 300 μg/mL G418 (Cat. G8168, Sigma-Aldrich) in RPMI 1640 (Cat. 61870044, Gibco) plus 10% fetal calf serum and 1 mM sodium pyruvate (Cat. 11360, Thermo Fisher Scientific). The clones were assayed for their ability to respond to tBHQ, a well-known NFE2L2 activator, by increasing luciferase production (Supplementary Fig. 1). For coculture experiments, 150,000 primary neuronal cells or 70,000 SK-N-BE or SK-ARE-*luc2* cells were plated in each well of a 24-well plate and cultured for 10 days (primary cells) or 1 day (cell lines). Then, primary microglia, BV-2 cells, MCF-7 cells, RAW 264.7 cells, or primary macrophages were seeded over the neuron layer: if not otherwise specified, 37,500 cells/well were seeded for primary microglia and primary macrophages, and 3500 cells/well were seeded for BV-2, RAW 264.7, and MCF-7 cells. For transwell experiments, 0.4-μm pore polyester membrane inserts (Cat. 3460, Corning) were used to separate BV-2 cells from adhered neuroblast cells. Neuronal and neuronal-microglial cocultures were grown in Neurobasal A medium (Cat. 10888-022, Life Technologies) containing 1% streptomycin–penicillin, 1% GlutaMAX, 2% B-27 Supplement (Cat. 17504-044; Gibco), and 10 mM HEPES (Cat. H0887, Merck) in a humidified 5% CO_2_/95% air atmosphere at 37 °C. Other cocultures were grown in RPMI 1640 medium containing FBS at a final concentration of 10%, 1% streptomycin–penicillin, and 1% GlutaMAX.

### Cell treatments

If not otherwise specified, cells were treated with 200 μM CBE or vehicle (water) for 48 h, with 1 μM cytochalasin D or vehicle (0.0005% v/v EtOH final) for 1 h, with 2 μM nocodazole or vehicle (0.02% v/v EtOH, 0.01% v/v DMSO final) for 2 h, and with 5 μM tBHQ, 15 μM tBHQ or vehicle (water) for 24 h.

### Flow cytometry assay

Flow cytometry experiments were performed on at least 200,000 cells for each sample by using a Novocyte 3000 (Agilent Technologies, Inc.) equipped with 488-nm lasers. Cells were incubated for 2 min at 4 °C with 0.1 mg/mL propidium iodide (Cat. P4170, Sigma-Aldrich), and fluorescence pulses were detected using a 585/40 nm collection filter. The results were analyzed using NovoExpress software (Agilent Technologies).

### Luciferase enzymatic assay

Luciferase assays were performed as described previously [28]. Briefly, cells were lysed with Luciferase Cell Culture Lysis Reagent (Cat. E1531, Promega), and the protein concentration was determined with a Bradford assay [32]. The biochemical luciferase activity assay was carried out in luciferase assay buffer by measuring luminescence emission with a luminometer (Veritas, Turner Biosystems), and the relative luminescence units (RLU) were determined during 10-second measurements.

### Glucocerebrosidase assay

A glucocerebrosidase assay was performed as previously described [21]. Cells were lysed with RIPA buffer, and the protein concentration was determined with BCA (Cat. 23227, Pierce). The biochemical assay of GCase activity was carried out in a buffer containing 4-methylumbelliferyl beta-d-glucopyranoside substrate (Cat. M3633, Sigma-Aldrich); after 1 h at 37 °C, the reaction was stopped with 0.25 M glycine buffer (pH 10.4), and the fluorescence emission of the 4-methylumbelliferyl generated by the reaction was read with a fluorimeter (EnSpire Plate Reader, PerkinElmer) with an excitation wavelength of 365 nm and an emission wavelength of 445 nm. The determined enzyme activity was expressed as the μmol 4-methylumbelliferyl generated in 1 h per μg of protein and normalized to the value in vehicle-treated cells.

### Immunofluorescence labeling

Mice were sacrificed via intraperitoneal (i.p.) injection of sodium pentobarbital and transcardially perfused with cold saline solution followed by 4% paraformaldehyde in PBS. The brains were immediately removed, postfixed in 4% paraformaldehyde for 24 h, and then cryopreserved in 30% sucrose. Serial coronal sections of 35 μm were cut throughout the brain using a freezing microtome. Free-floating sections were rinsed in Tris-HCl (pH 7.6), and nonspecific binding sites were blocked by incubation in Tris-buffered saline solution containing 0.25% Triton X-100 and 5% normal horse serum at room temperature (RT) for 1 h. The sections were kept for two nights at 4 °C in Tris-buffered saline with 1% BSA, 0.25% Triton X-100, and the following primary antibodies: mouse anti-tyrosine hydroxylase (Th) (1:300; Cat. MAB318, Chemicon) and rabbit anti-Nfe2l2 (1:300; Cat. AB3116, Abcam). They were then rinsed and incubated with a DyLight 594-conjugated horse anti-rabbit secondary antibody (1:300, Vector Laboratories) and a DyLight 649-conjugated horse anti-mouse antibody (1:300, Vector Laboratories) for 1 h at 4 °C. Finally, the samples were counterstained with DAPI (1:10,000 in Tris-HCl, Thermo Fisher Scientific) and mounted with a coverslip using VECTASHIELD mounting medium (Vector Laboratories). Fluorescence stack images were collected at 1.29-μm intervals throughout the midbrain using Zeiss microscopes (LSM880) with a 40× Plan-Apochromat objective.

Cocultures of SK-N-BE and BV-2 cells grown in a 24-well chamber were fixed in 4% paraformaldehyde fixative for 15 min at RT and washed three times for 5 min with PBS [33]. The fixed cells were incubated at RT for 1 h in blocking solution containing 0.1% p/v BSA (Cat. A9418, Merck), 10% v/v goat serum (Cat. ECS0200D, Euroclone), and 0.1% v/v Triton X-100 (Cat. T-9284, Sigma). Next, the cells were incubated at 4 °C with 10% blocking solution in PBS with a rat anti-CD11b antibody (diluted 1:500; Cat. MCA7114, Serotec) and a rabbit anti-NFE2L2 antibody (diluted 1:500; Cat. C-20, Santa Cruz Biotechnology). After 24 h, the fixed cells were rinsed three times with PBS and incubated for 1 h at RT in 10% blocking solution composed of PBS with a mixture of an Alexa Fluor 488-conjugated goat anti-rat IgG antibody (diluted 1:300, Cat. A-11006, Molecular Probes Inc.) and an Alexa Fluor 555-conjugated goat anti-rabbit IgG antibody (diluted 1:300; Cat. A-21429, Molecular Probes Inc.). Finally, the fixed cells were rinsed in PBS and covered with 1:1000 DAPI solution in PBS (Cat. D1306, Thermo Fisher Scientific). After extensive washing, image acquisition was performed for 20 random fields per condition using an Axiovert 200M microscope with dedicated software (AxioVision Rel 4.9, Zeiss).

### Immunofluorescence analysis

For the brain slices, semiquantitative analysis of Nfe2l2 nuclear intensity was performed within neurons of the substantia nigra pars compacta (SNpc). For each animal, two SNpc-containing midbrain sections were analyzed. Sections were collected at similar anatomical levels and processed with Fiji software (ImageJ, NIH, version 2.0.0). The software selected and delineated DAPI-stained nuclear areas. Within these areas, the Nfe2l2 gray values were measured and averaged.

For coculture, the fluorescence images were processed with Fiji software (ImageJ, NIH, version 2.0.0). In brief, the regions of interest (ROIs) of cellular nuclei automatically generated by the software using the DAPI field were utilized to quantify the average gray value of the NFE2L2 field. The value related to the nuclei of Cd11b+ cells was manually excluded from the analysis.

### Animal treatments

The animals were fed ad libitum and housed in individually ventilated plastic cages within a temperature range of 22–25 °C under a relative humidity of 50% ± 10% and an automatic cycle of 12 hours light/dark (lights on at 07:00). ARE-*luc2* mice were generated in our laboratory [28]. For pharmacological treatments, mice (15–30 weeks old) were administered (i) 100 mg/kg/day CBE or vehicle (PBS) via i.p. injection for 3 days, (ii) 75 mg/kg tBHQ dissolved in PBS + 1% DMSO + 20% PEG300 via i.p. injection, or (iii) 100 μg of PLX3397 or vehicle solution (5% DMSO + 45% PEG300 + ddH2O) in nose drops (3 μL/drop; one drop in each nostril, corresponding to 6 μL/administration, with alternation between the left and right nostrils twice at intervals of 2 min; subsequent doses were given every 12 h for 1 week). For transnasal administration, anesthetized mice were placed in the supine position, and a heated pad was inserted under the dorsal neck to induce hyperextension of the neck (tilting the head backward) [34].

### In vivo and ex vivo imaging

For semiquantitative analysis of photon emission, animals were injected subcutaneously with 80 mg/kg luciferin 15 min prior to the imaging session. The mice were anaesthetized using isoflurane and kept under anesthesia during each 5 min optical imaging session carried out with a charge-coupled device (CCD) camera (IVIS Lumina II Quantitative Fluorescent and Bioluminescent Imaging, PerkinElmer). Photon emission in different brain areas was measured using Living Image Software v. 4.2 (PerkinElmer). The mice were killed by cervical dislocation after the last in vivo acquisition, and the brains were rapidly dissected and sectioned by means of a “brain matrix” (adult mouse, coronal and sagittal, 1 mm spacing). The sections were immediately subjected to a 5-min ex vivo imaging session. Photon emission was quantified with Living Image Software v. 4.2.

### Real-time PCR (RT-PCR)

RT-PCR analyses were performed as previously described [28]. In brief, total RNA was extracted from BV-2 cells using a Direc-zol RNA Miniprep kit (Cat. R2050, Zymo Research), and cDNA synthesis was performed using Moloney murine leukemia virus reverse transcriptase (Cat. M3681, Promega) and random primers (Cat. C118A, Promega) [35]. A mass of 0.5 μg of RNA was denatured at 70 °C for 5 min in the presence of 0.75 μg of random primers in a 7.5-μL final volume. RT-PCR was carried out at 37 °C for 1 h, and the enzyme was inactivated at 75 °C for 5 min. For each sample, control reactions were routinely performed without addition of reverse transcriptase. A 1:20 cDNA dilution was amplified using SYBR Green chemistry in triplicate in a 96-well plate using GoTaq qPCR Master Mix technology (Cat. A6001, Promega) according to the manufacturer’s protocol (5 μL of qPCR master mix, 0.15 μL of 100 mM primers each) and 4.7 μL of cDNA) using a QuantStudio 3 - 96-Well 0.1 mL Block (Thermo Fisher Scientific) with the following thermal profile: 2 min at 95 °C and 40 cycles of 15 s at 95 °C and 1 min at 60 °C. The primers used are listed in Table 1 (Eurofins), and quantification was performed using the comparative CT method (2^−ΔΔCt^).

**Table 1:** Primers used for RT-PCR

Gene	Forward (5′-3′)	Reverse (5′-3′)
*Rplp0*	GGCGACCTGGAAGTCCAACT	CCATCAGCACCACAGCCTTC
*Nfe2l2*	CCCAGCAGGACATGGATTTGA	AGCTCATAGTCCTTCTGTCGC
*Arg1*	CAGAAGAATGGAAGAGTCAG	CAGATATGCAGGGAGTCACC
*Trem2*	GGAACCGTCACCATCACTCT	CTTGATTCCTGGAGGTGCTGT
*Il-1b*	TGCCACCTTTTGACAGTGATG	GCTGCGAGATTTGAAGCTGG
*Nqo1*	GGTAGCGGCTCCATGTACTC	CGCAGGATGCCACTCTGAAT
*Tfeb*	CCAGAAGCGAGAGCTCACAGAT	TGTGATTGTCTTTCTTCTGCCGC
*Lamp1*	GCCCTGGAATTGCAGTTTGG	TGCTGAATGTGGGCACTAGG
*Cx3cr1*	CTGCTCAGGACCTCACCATG	CACCAGACCGAACGTGAAGA
*P2ry12*	GAACCAGGACCATGGATGTG	CCAAGCTGTTCGTGATGAGC
*C3*	GGAAGATCCGAGCCTTTTAC	CCACACCATCCTCAATCACTAC

### Statistical analysis

Unless otherwise indicated in the figure legend, variables are presented as the mean with standard deviation. Statistical analyses were performed using Prism 7 (Version 7.00, GraphPad Software Inc.). *T*-tests were used to determine if there were significant differences in means between two groups. One-way ANOVA was used to determine if there were statistically significant differences in means among three or more independent groups; post hoc Tukey’s test was used to compare every mean with every other mean, or Dunnett’s test was used to compare every mean to a control mean [36]. Two-way ANOVA followed by Sidak’s post hoc test was used to determine if the responses were affected by two factors in a multiple comparison. A *p*-value lower than 0.05 was considered to indicate statistical significance.

## Results

### GCase inhibition impairs the Nfe2l2 response in vivo

**Fig. 1:**
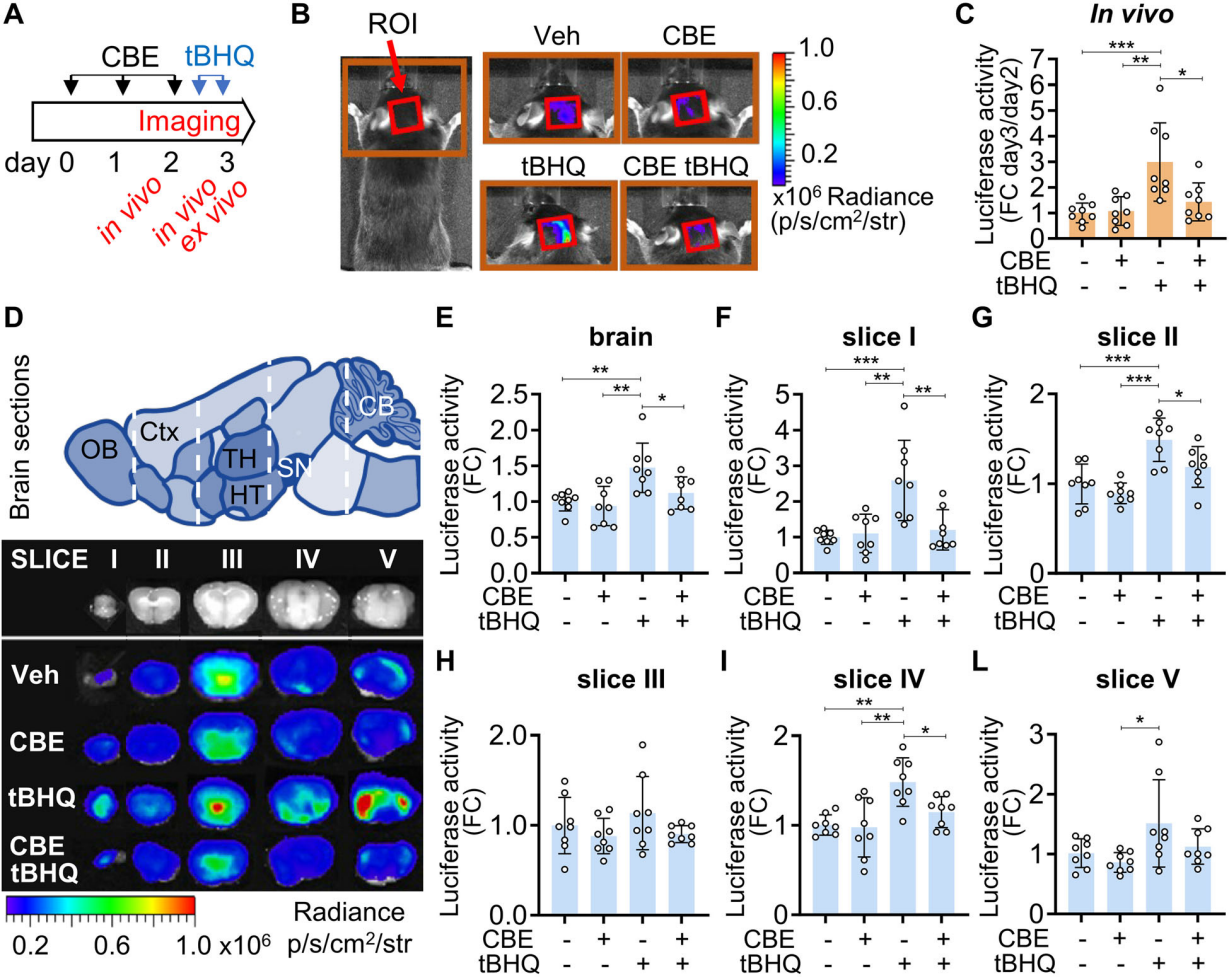
Bioluminescence analysis of Nfe2l2 activation in ARE-*luc2* reporter mice treated with CBE**. A** Schematic representation of the experiments, reporting the timing of the pharmacological treatments and the in vivo/ex vivo imaging sessions. **B** Representative pictures of the in vivo bioluminescence detected in the selected regions of interest (ROI, red square) in the head area: the bioluminescence signal is shown as radiance photons (p/s/cm^2^/sr) and represented as pseudocolors, according to the reported scale bar. **C** Bioluminescence imaging (BLI) quantifications of the photon emission from the ROI shown in **B**; the measures of bioluminescence signal are reported in the graph as fold change (FC) of the radiance photons measured at the end of the pharmacological treatment (day 3) *versus* the radiance photons measured before the treatment with tBHQ (day 2). **D** Representative ex vivo bioluminescence imaging of the brain dissected in five sections (slices I–V) and the schematic representation of the different brain areas in each slide. CB, cerebellum; Ctx, cerebral cortex; HT, hypothalamus; OB, olfactory bulb; SN, substantia nigra; TH, thalamus. Bioluminescence signals were acquired for each brain section at the end of the pharmacological treatments and are shown as radiance photons (p/s/cm^2^/sr) and represented as pseudocolors, according to the reported scale bar. Quantification of the BLI signals from whole brain and brain slices are reported in **E** and **F**–**L**, respectively: the measures of bioluminescence signal are reported in the graph as fold change (FC) of the radiance photons *versus* vehicle and presented as mean ± SD of *n*=4 independent samples measured in duplicate. Statistical significance was determined by one-way ANOVA followed by Tukey’s multiple comparison test. *p<0.05, **p<0.01, ***p<0.001

Two separate sets of in vivo experiments were carried out to investigate potential interactions between GCase activity and the Nfe2l2 pathway. First, changes in the Nfe2l2-dependent oxidative stress response caused by GCase inhibition were assessed by in vivo and ex vivo imaging of luciferase activity in the brains of ARE-*luc2* reporter mice (Fig. 1). In these transgenic animals, the luciferase reporter is expressed under the control of the Nfe2l2 transcription factor [28, 37]. Two groups of 16 reporter mice were treated with a daily i.p. dose of 100 mg/kg/day CBE or vehicle (PBS) for 3 days. This dose was sufficient to decrease GCase activity to 50% of baseline levels (Supplementary Fig. 2A); the same reduction has been observed in fibroblasts obtained from heterozygous patients carrying severe GBA mutations [5]. On day 2, half of the mice from both groups were further treated with two i.p. injections (20 and 16 h prior to the end of the experiment) of 75 mg/kg tBHQ, a well-known Nfe2l2-inducing agent that crosses the blood-brain barrier [28, 38] (Fig. 1A). Bioluminescence emissions were measured before and after tBHQ administration by in vivo imaging (Fig. 1A). Quantitative analysis of photon emission from the head areas of CBE- and vehicle-treated mice revealed comparable luciferase activity, indicating that CBE administration did not affect the physiological Nfe2l2 signaling pathway (Fig. 1B, C). tBHQ treatment induced a significant (threefold) increase in luciferase activity in vehicle-treated animals. Remarkably, however, this Nfe2l2 response was impaired in mice pretreated with CBE. At the end of these experiments, brains were collected and dissected, and *ex vivo* imaging was carried out on whole brains as well as on 2- to 3-mm-thick brain slices (Fig. 1D). In line with the in vivo results, quantification of luciferase activity showed that the increase after administration of tBHQ was attenuated in CBE-treated animals. This effect reached statistical significance in the whole brain (Fig. 1E) and in brain slices I (−53%), II (−20%), and IV (−21%) (Fig. 1F, G, I).

**Fig. 2:**
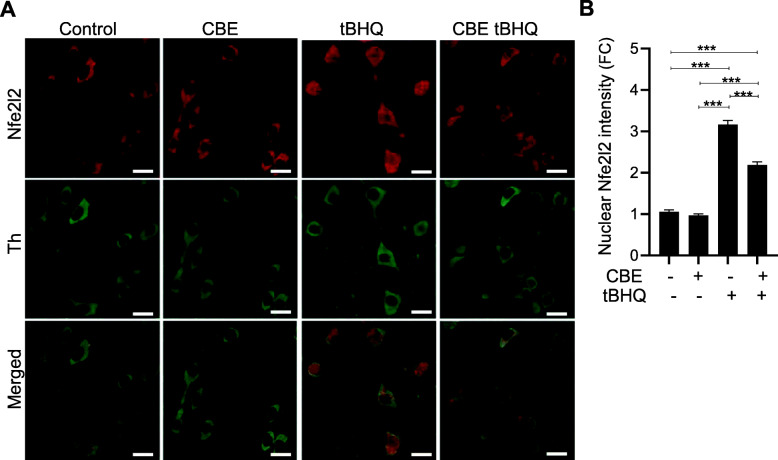
Nfe2l2 activation in nigral dopaminergic neurons. Wild-type mice (C57BL/6 strain, *n* = 4/group) were treated as reported in Fig. 1A. **A** Representative confocal images of the substantia nigra pars compacta. Midbrain sections immunostained with anti-Nfe2l2 (red) and anti-Tyrosine hydroxylase (Th) (green) antibodies. Scalebar, 10 μm. **B** Semi-quantitative analysis of Nfe2l2-associated fluorescence in the nuclei of nigral dopaminergic neurons. Data are expressed as fold changes (FC) relative to the control value and presented as mean ± SEM. Statistical significance was determined by one-way ANOVA followed by Tukey’s post hoc test. ***p<0.001

Next, wild-type C57BL/6 mice were divided into 4 experimental groups and treated with vehicle, CBE, tBHQ, or CBE plus tBHQ as described above. Postmortem analyses were performed on tissue sections of the ventral mesencephalon that contained the SNpc and were immunostained with anti-Th and anti-Nfe2l2 antibodies. Immunoreactivity for Nfe2l2 (red staining) was detected within the cytosolic compartment in Th-positive nigral dopaminergic neurons in sections from vehicle- and CBE-treated mice (Fig. 2A). In contrast, Nfe2l2 staining became mostly nuclear after tBHQ administration, consistent with nuclear translocation of this transcription factor. Nfe2l2 nuclear immunoreactivity in the SNpc was apparently less robust in animals injected with both CBE and tBHQ than in animals injected with tBHQ alone (Fig. 2A). Semiquantitative analysis of nuclear Nfe2l2 intensity confirmed these histochemical observations. There was a 3-fold increase in nuclear Nfe2l2 after treatment with tBHQ alone and a significant reduction (−30%) in this effect in mice with CBE-induced GCase inhibition (Fig. 2B). Taken together, the results of these in vivo experiments provide the first evidence of a relationship between GCase activity and Nfe2l2 transcriptional activity, suggesting that impairment of GCase alters neuronal antioxidant responses in different brain regions, including the SNpc. Follow-up studies were then designed to investigate the mechanisms underlying this relationship.

### GCase inhibition hinders the microglia-mediated induction of NFE2L2 signaling in neurons

**Fig. 3:**
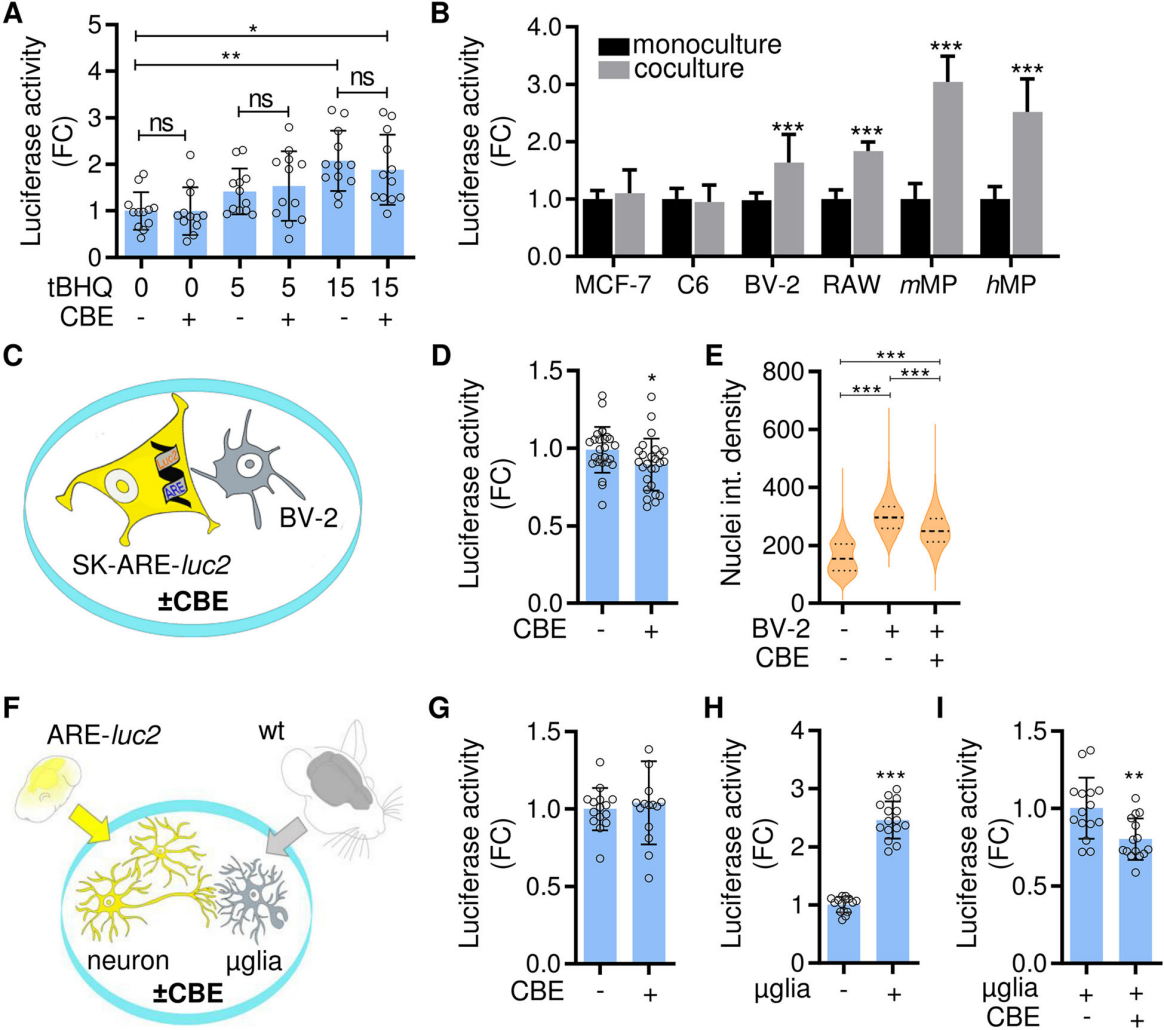
CBE treatment reduces the neuronal NFE2L2 response induced by microglia. **A** The NFE2L2 activity was measured in SK-ARE-*luc2* cells treated with vehicle (water) or 200 μM CBE for 48 h, then treated with vehicle or 5 μM and 15 μM tBHQ for 24 h. Data are expressed as fold changes (FC) of RLU/μg protein extracts, *versus* vehicle and presented as mean ± SD of *n*=4 independent samples measured in triplicate. Statistical significance was determined by one-way ANOVA followed by Dunnet’s multiple comparison test *versus* vehicle and unpaired *t*-test between CBE or vehicle in equal concentration of tBHQ. ns, not significant, *p<0.05, **p<0.01. **B** The NFE2L2 activity was measured in SK-ARE-*luc2* cocultured for 48 h with different cell lines (MCF-7; C6; BV-2; RAW 249.7) and primary cultures (murine peritoneal macrophages, *m*MP); human macrophages, *h*MP). Data are expressed as fold changes (FC) of RLU mean values *versus* SK-ARE-*luc2* monoculture and presented as mean ± SD for *n*=9 independent samples measured in triplicate. Statistical significance was determined by two-way ANOVA followed by Sidak’s multiple comparisons test. ***p<0.001. **C** Scheme of the experiments reported in **D** and **E** aimed at testing the effect of CBE on neuronal NFE2L2 activation in SK-ARE-*luc2*/BV-2 cocultures. **D** Data are expressed as fold changes (FC) of RLU *versus* vehicle and presented as mean ±SD of *n*=9 independent samples measured in triplicate. *p<0.05, ***p<0.001 calculated by unpaired *t*-test. **E** Data report the semiquantitative analysis of the average fluorescence of SK-ARE-*luc2* nuclei, following Nfe2l2 immunostaining, and presented as violin-plot with median (dash line) and quartiles (dot lines) of the analysis of *N*=6000 nuclei measured in 20 fields/group (see Supplementary Fig. 4). Statistical significance was determined by one-way ANOVA followed by Tukey’s multiple comparison test. ***p<0.001. **F** Scheme of the experiments reported in **G**–**I**, aimed at testing the effects of CBE in primary cocultures of neurons and microglia (μglia). **G** Luciferase activity from ARE-*luc2* primary neurons treated with 200 μM CBE for 48 h in monoculture, or cocultured with wild type primary microglia. ARE-*luc2* primary neurons cocultured with wild type primary microglia treated with vehicle (**H**) or with 200 μM CBE for 48 h (**I**). Data are expressed as fold changes (FC) of RLU *versus* vehicle treated samples (**G**), *versus* neuron monoculture (**H**) and *versus* vehicle treated cocultures (**I**), and presented as mean ±SD of *n*=5 independent samples measured in triplicate. *p<0.05, ***p<0.001 calculated with the unpaired *t*-test

To characterize the mechanism of GCase inhibition, we investigated the effect of CBE in neuronal cells and in microglia/neuron cocultures. Initial experiments were carried out in SK-N-BE dopaminergic neuroblastoma cells [39] stably transfected with the bioluminescent NFE2L2 activity reporter pARE-*luc2*-ires-tdTomato [37], named SK-ARE-*luc2*. Cells were treated with 200 μM CBE or vehicle for 48 hours to ensure that GCase activity was inhibited (Supplementary Fig. 2B) with negligible effects on the activity of additional glycosidase targets [40]. At 24 h before harvest, cells were also treated with tBHQ at different concentrations (5 and 15 μM). The enzymatic quantification of luciferase in protein extracts demonstrated a concentration-dependent increase in reporter activity in tBHQ-treated cells. However, in contrast with the in vivo observation (Fig. 1), CBE treatment did not affect the tBHQ-induced NFE2L2 activation (Fig. 3A), suggesting that GCase impairment in neuronal cells does not modulate NFE2L2 activity. Previous studies have shown that brain cells other than neurons cooperate to regulate oxidative stress responses in Parkinson’s disease-associated neurodegeneration [28, 41] and are able to provide “clue” signals that in turn induce the NFE2L2 pathway in neuronal cells. Therefore, we used microglial BV-2 and glioma C6 cell lines to perform coculturing experiments with SK-ARE-*luc2* cells. Interestingly, the presence of BV-2 cells per se, but not C6 cells, increased neuronal NFE2L2 activity; this effect was also induced by other cells of macrophage/monocyte lineages (RAW 264.7 cells and mouse and human primary macrophages) but not cells of other lineages (MCF-7 cells) (Fig. 3B). We next experimentally determined the optimal ratio between BV-2 and SK-ARE-*luc2* to produce the highest NFE2L2 activation and found that 1:20 was the optimal composition (+60% NFE2L2 activity). This ratio closely represents the physiological neuron/microglia ratio in the brain [42] (Supplementary Fig. 3). The effect was not due to peculiar features of the transformed cell lines since a 250% increase in NFE2L2 activity was also observed in cocultures of primary mouse microglia and neurons. The latter were obtained from ARE-*luc2* reporter mice and thus carried the luciferase reporter system for the NFE2L2 pathway (Fig. 3H). These data show that microglia/macrophages communicate with neuronal cells to induce neuronal NFE2L2 activity. Next, we tested the effects of specific inhibition of microglial GCase on neuronal NFE2L2 activity. Although treatment with 200 μM CBE did not significantly reduce the basal level of NFE2L2 activity (Fig. 3A, G), it significantly inhibited the microglia-mediated induction of the neuronal NFE2L2 pathway in both transformed lines (−10%) (Fig. 3D) and primary cells (−20%) (Fig. 3I). The observed inhibitory effect was greater in primary cultures than in cell lines, indicating that microglia-induced activation of the neuronal NFE2L2 pathway is more effective in naïve cells. Finally, these results were confirmed by semiquantitative analysis of the fluorescence intensity in immunohistochemistry experiments with an anti-NFE2L2 antibody. Increased nuclear localization of NFE2L2 was observed in SK-N-BE cells when the cells were cocultured with the microglial cell line, but this effect less marked when the cocultures were treated with CBE (Fig. 3E, Supplementary Fig. 4).

### Microglial GCase is essential for the efficient activation of the NFE2L2 pathway in neurons

**Fig. 4:**
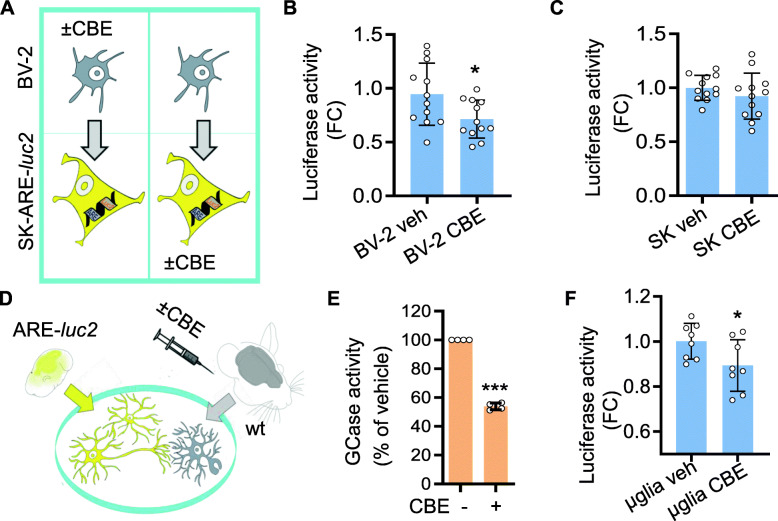
Inhibition of microglial GCase hamper the neuronal Nfe2l2 response. **A** Schematic representation of the experiments summarized in (**B**, **C**) to assess the effect of the selective GCase inhibition in microglial or neuronal cells. BV-2 (**B**) or SK-ARE-*luc2* (**C**) cells were pre-treated with 200 μM CBE for 48 h before seeding the cocultures. Data are expressed as fold changes (FC) of RLU *versus* vehicle and presented as mean ±SD of *n*=4 independent samples measured in triplicate. *p<0.05 by unpaired *t*-test. **D** Schematic representation of the experiments reported in **E** and **F** aimed at testing the effect of primary microglia extracted from mice treated with 100 mg/kg/die CBE for 3 days. **E** GCase activity in the primary microglia expressed as % of the activity detected in microglia extracted from the vehicle treated animals. Data are presented as mean ±SD of *n*=2 independent samples measured in duplicate. ***p<0.001 calculated by unpaired *t*-test. **F** Luciferase activity measured in protein extracts derived from ARE*-luc2* neurons cocultured with microglia derived from CBE or vehicle treated mice. Data are presented as mean ±SD of *n*=4 independent samples measured in duplicate. *p<0.05 calculated by unpaired *t*-test.

**Fig. 5:**
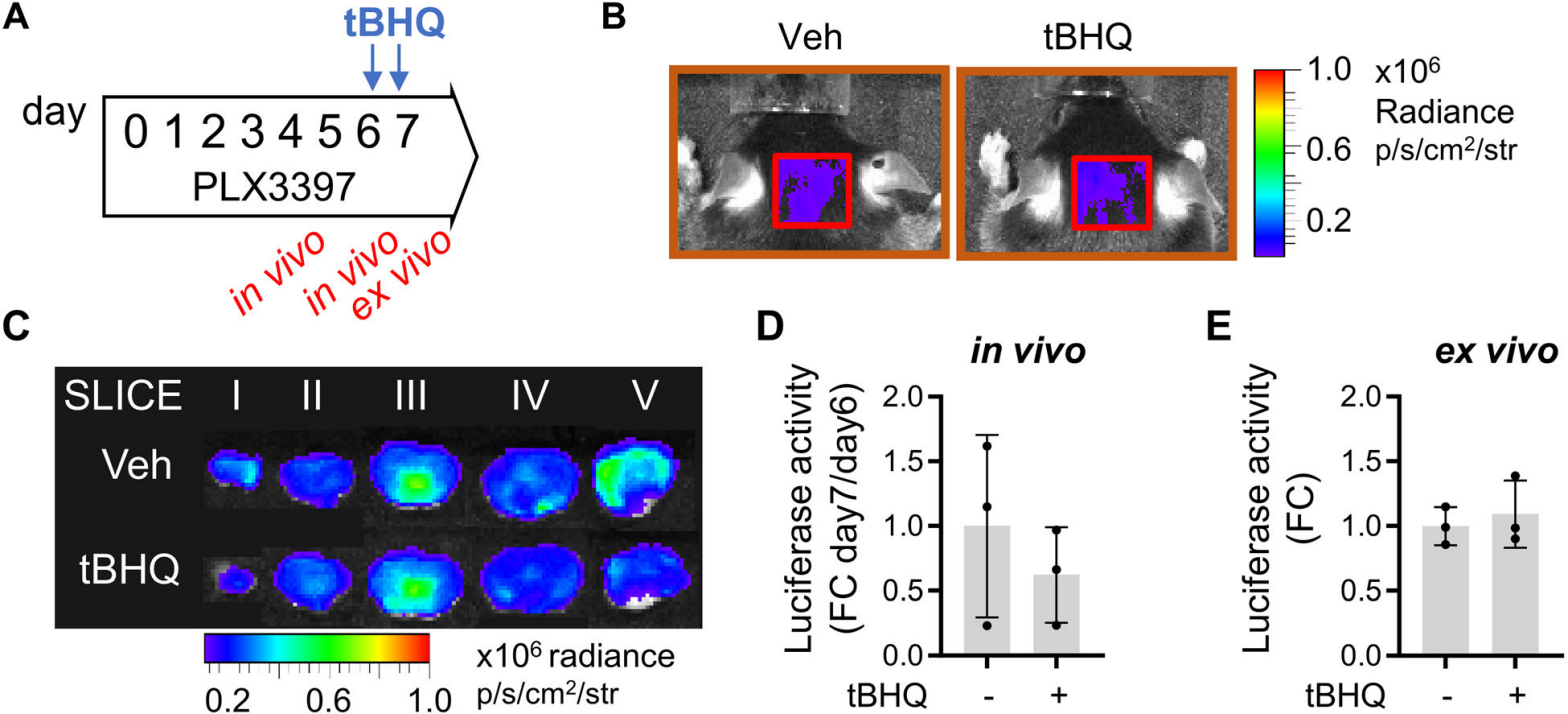
Nfe2l2 response in vivo is microglia-dependent. **A** Schematic representation of the experiment: mice underwent pharmacological depletion of microglia with 100 μg PLX3397 administered every 12 h for 1 week: 20 and 16 h before the end of the experiment mice received two i.p. injection of 75 mg/kg tBHQ. Bioluminescence Imaging (BLI) was acquired from the brain area (see ROI Fig. 1B) or from the dissected brain for each mouse by in vivo (**B**) and ex vivo imaging (**C**). Representative acquisitions are shown as radiance photons (p/s/cm^2^/sr) and represented as pseudocolors, according to the reported scale bar. The measures of bioluminescence signal are reported in the graphs (**D**) and (**E**) as fold change (FC) of the radiance photons versus the value obtained before tBHQ administration (day 6) (for the in vivo imaging) (**D**) or on the value obtained from the vehicle treated mice (for *ex vivo* imaging) (**E**). Data are presented as mean ±SD of *n*=3 independent samples. Differences were not significant, by unpaired *t*-test

To test the hypothesis that the CBE-mediated downregulation of neuronal NFE2L2 activity observed in vivo could be ascribed to microglial GCase inhibition, either BV-2 or SK-ARE-*luc2* cells were pretreated with 200 μM CBE for 48 h before the coculture was seeded (Fig. 4A). Luciferase activity in protein extracts decreased significantly (−25%) only when BV-2 cells were pretreated with CBE (Fig. 4B, C), demonstrating that inhibition of microglial GCase was necessary and sufficient to reduce neuronal NFE2L2 activity in the coculture. This conclusion was confirmed in primary cells, i.e., microglia obtained from mice previously administered 100 mg/kg/day CBE for 3 days or vehicle (PBS) via i.p. injection and cultivated for 24 h on primary neuronal cells derived from ARE-*luc2* mice (Fig. 4D, F). The GCase activity was halved in microglia obtained from CBE-treated mice (Fig. 4E), the NFE2L2 activation was reduced by 20% in primary ARE-*luc2* neurons (Fig. 4F) compared to microglial cocultures obtained from vehicle-treated mice. The key role played by microglia in neuronal NFE2L2 activation was firmly demonstrated through pharmacological depletion experiments using PLX3397, a colony stimulating factor-receptor 1 inhibitor [43]. Microglia were depleted in ARE-*luc2* mice after seven intranasal twice-daily administrations of 100 μg of PLX3397 [29]. Half of the treated mice also received two i.p. doses of 75 mg/kg tBHQ at 20 and 16 h before the end of the experiment to induce the NFE2L2 pathway (Fig. 5A). In vivo and ex vivo imaging-based quantification of the photon emission from the head areas and from the dissected brains of the ARE-*luc2* mice (Fig. 5B, C) showed that depletion of microglia hindered the induction of the Nfe2l2 pathway observed upon tBHQ administration (Fig. 5D, E). Taken together, these data demonstrate that the neuronal Nfe2l2 response in the brain requires the presence of functional microglia.

### GCase inhibition hampers the neuroprotective functions of microglia

**Fig. 6:**
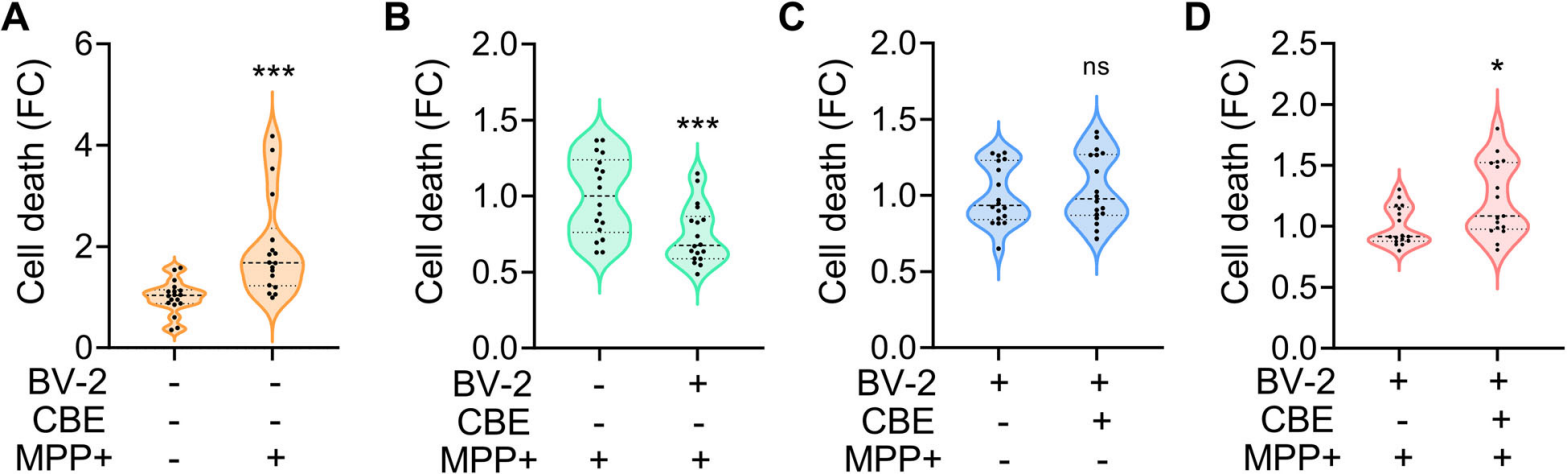
GCase inhibition hampers neuroprotective functions of microglia. Flow cytometry analyses of propidium iodide fluorescence in: **A** SK-N-BE treated with 0.5 mM MPP+ or vehicle for 24 h, **B** SK-N-BE cultured alone or in coculture with BV-2 and treated with 0.5 mM MPP+, **C** cocultures treated with 200 μM CBE for 48 h or **D** with 200 μM CBE for 48 h and 0.5 mM MPP+ for 24 h. Data are expressed as fold changes (FC) *versus* vehicle treated samples (**A**, **C**, **D**) or monoculture sample (**B**), and presented as violin-plots with median (dash line) and quartiles (dot lines) of *n*=6 independent samples measured in triplicate.*p<0.05, ***p<0.001 calculated by unpaired *t*-test

The NFE2L2 pathway is critical for detoxification after oxidative insult; thus, we tested whether microglia-induced activation of the neuronal NFE2L2 pathway was sufficient to increase the detoxification capability of neurons. Microglia-induced neuroprotection was tested in SK-N-BE neuroblastoma cells treated with 0.5 mM MPP+, the active metabolite of MPTP that blocks the mitochondrial electron transport enzyme NADH:ubiquinone reductase (H(+)-translocating) [44, 45] and induces cellular death as a consequence of reactive oxygen species formation [46]. This treatment doubled the cell death in the neuroblastoma cell monoculture (Fig. 6A, Supplementary Fig. 5), as quantified by flow cytometry analysis of the propidium iodide-stained dead cells. Cell death was attenuated by 25% when the neuroblastoma line was cocultivated with BV-2 microglial cells (Fig. 6B), confirming that microglia were able to increase neuronal resistance to oxidative insults. Conversely, microglia-dependent neuroprotection was blunted when GCase was inhibited with 200 μM CBE for 48 h (Fig. 6D), while CBE treatment alone did not affect cell viability in the absence of neurotoxic stimulation (Fig. 6C). These data demonstrate that GCase inhibition hampers the neuroprotective functions of microglial cells.

### Inhibition of microglial GCase increases the expression of genes associated with neurodegeneration

**Fig. 7:**
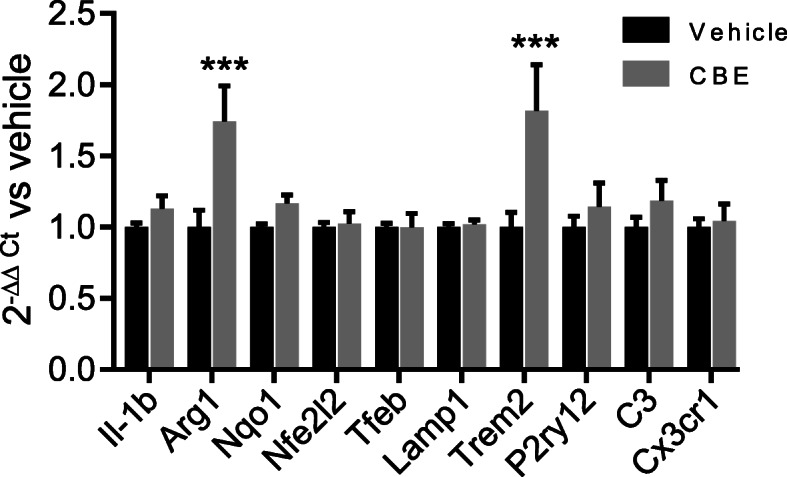
Modulation of gene expression by GCase inhibition in BV-2 cells. Total RNA was purified from BV-2 cells treated with 200 μM CBE for 48 h and the expression of selected mRNA were analysed by real-time PCR. Relative quantification of the transcript was obtained using the 2^−ΔΔCt^ method versus the vehicle-treated samples. Data are presented as mean ±SD of *n*=3 independent samples measured in triplicate. Statistical significance was determined by two-way ANOVA followed by Sidak's multiple comparisons test. ***p<0.001

To gain molecular insights into the mechanism of neuroprotection, we measured the expression of genes previously associated with specific microglial phenotypes via real-time PCR analysis of mRNA purified from BV-2 cells treated with 200 μM CBE or vehicle for 48 h (Fig. 7). We did not observe increased expression of the proinflammatory cytokine interleukin 1 beta (*Il-1b*) or altered regulation of Nfe2l2 target genes (*Nqo1* and *Nfe2l2*), confirming that GCase inhibition did not trigger an inflammatory phenotype [47] or alter antioxidant and detoxification response regulation in microglia. Surprisingly, the expression of genes involved in the lysosomal pathway (*Lamp1* and *Tfeb*) and genes involved in intercellular communication and selective phagocytosis (*Cx3cr1*, *C3* and *P2ry12*) [10, 48] were unaffected, suggesting that microglia maintained their homeostatic functionality [49, 50]. Interestingly, two different genes involved in anti-inflammatory and repair pathways, *Arg1* and *Trem2* [51], were upregulated following microglial GCase inhibition. In particular, the latter, which is also involved in microglial metabolic reprogramming, is a disease-associated gene whose mutations are linked with the onset of neurodegenerative diseases such as Alzheimer’s disease [52] and Parkinson’s disease [53]. Recent reports have proposed that Trem2 acts as the principal regulator triggering a specific microglial phenotype associated with neural diseases by regulating the ability of microglia to phagocytose debris [54].

These data suggest that GCase represents a key node in the regulation of microglial neuroprotective activity. This node ensures the activation of detoxification/oxidative stress pathways in neurons and modulates a reparative/anti-inflammatory microglial response to counteract neurodegenerative insult.

### Microglia require functional actin-dependent structures to induce NFE2L2 in neuronal dopaminergic cells

**Fig. 8:**
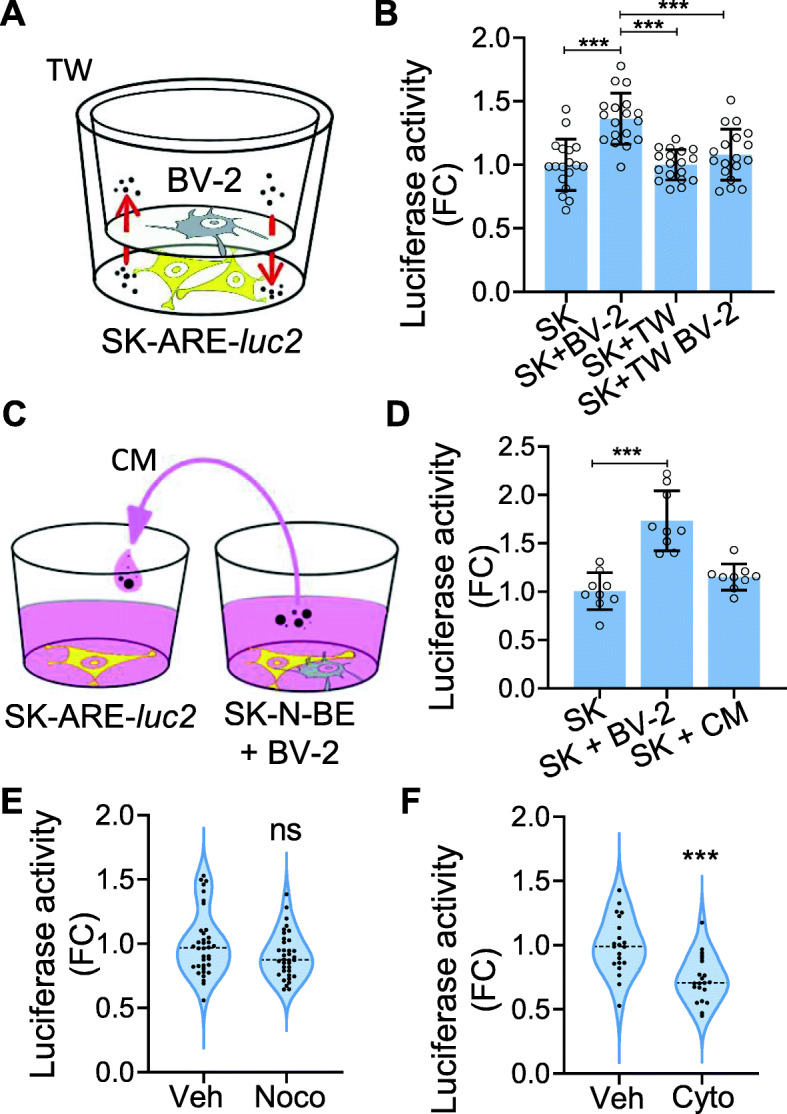
Mechanism of microglia-to-neuron cross-talk. **A** Scheme of the transwell (TW) settings used in the experiment reported in **B**: BV-2 and SK-ARE-*luc2* cells were seeded in separate compartment of the TW or in direct contact. **B** Luciferase enzymatic activity obtained from coculture of SK-ARE-*luc2* and BV-2 cells (SK+BV-2), or SK-ARE-*luc2* growth alone in the transwell (SK+TW), or growth in separated compartment with microglia (SK+TW+BV-2); luciferase activity is expressed as fold change (FC) *versus* the RLU value measured for the monocultures of SK-ARE-*luc2* (SK) and presented as mean ±SD of *n*=6 independent samples measured in triplicate. Statistical significance was determined by one-way ANOVA followed by Tukey’s multiple comparison test. ***p<0.001. **C** Schematic representation of the experiment reported in **D**: SK-ARE-*luc2* cells were grown in coculture with BV-2 (SK+BV-2) or in monoculture with the conditioned medium (SK+CM) obtained from the coculture. **D** Luciferase activity (RLU) is expressed as FC *versus* the value measured for the SK-ARE-*luc2* monocultures (SK) and presented as mean ±SD of *n*=3 independent samples measured in triplicate. Statistical significance was determined by one-way ANOVA followed by Dunnet’s multiple comparison test *versus* SK. ***p<0.001. **E** BV-2 were pre-treated with 25μM nocodazole (noco) or vehicle for 2 h and cocultured SK; luciferase activity is expressed as fold changes (FC) *versus* the RLU value measured for the vehicle (veh) and presented as violin-plot with media (dashed lines) and quartiles (dotted lines) of *n*=12 independent samples measured in triplicate. Mean differences are not significant as calculated by unpaired *t*-test. **F** BV-2 were pre-treated with 1μM cytochalasin (cyto) or vehicle (veh) for 1 h and cocultured with SK-ARE-*luc2*; luciferase activity is expressed as fold changes (FC) *versus* the RLU value measured for the vehicle and presented violin-plot with median (dash line) and quartiles (dot lines) ±SD of *n*=7 independent samples measured in triplicate

Finally, we investigated whether microglia-to-neuron communication that induced NFE2L2 activity required a paracrine mechanism based on the release of secreted factors or a physical interaction between cells [55–57]. For this purpose, BV-2 and SK-ARE-*luc2* cells were grown in separate compartments (created using a 0.4 μm membrane) of the same well (Fig. 8A), which prevented physical interactions between the two cell types while preserving the exchange of soluble factors. Under these conditions, neuronal NFE2L2 induction was not observed (Fig. 8B), suggesting that this response does not require diffusible factors. To exclude the possibility that the membrane pore of 400 nm obstructed the diffusion of larger macromolecular complexes (e.g., extracellular vesicles), conditioned media derived from cocultured BV-2 and SK-N-BE cells were added to SK-ARE-*luc2* cells (Fig. 8C). Again, induction of neuronal NFE2L2 activity was not observed (Fig. 8D), thus definitely ruling out a possible role of microglia-secreted factors in this phenomenon. These experiments prompted us to test the alternative hypothesis of a mechanism based on the direct contact between the two cell lineages. Microglia use two major types of branches that are strictly regulated by distinct intracellular signaling cascades to sense the environment: actin-dependent filopodia and tubulin-dependent large processes [56, 58–60]. To determine which of the two pathways is required for the microglia-dependent induction of NFE2L2 activity, BV-2 cells were pretreated with 25 μM nocodazole, an agent preventing microtubule assembly, for 2 h. This concentration and duration are known to inhibit large processes without affecting filopodia movement [59]. After the treatment, cells were seeded on SK-ARE-*luc2* cells, and luciferase activity was measured 24 h later. The results demonstrated that pharmacological disruption of microglial large processes did not affect the ability of microglia to induce NFE2L2 signaling in neurons (Fig. 8E). In contrast, when BV-2 cells were pretreated with 1 μM cytochalasin for 1 h to block actin polymerization and reduce filopodia motility [59, 61], the induction of neuronal NFE2L2 activity was significantly decreased (>25%) (Fig. 8F), suggesting that actin-dependent structures are necessary for the microglia-mediated stimulation of the NFE2L2 pathway in neurons.

## Discussion

GBA mutations are very strongly correlated with Parkinson’s disease, indicating that dysfunction of this gene is one of the major risk factors for development of this neurodegenerative disease [5]. Numerous mechanisms have been proposed to explain the GBA-associated neurodegenerative phenotype, although most related studies have focused on both gain- and loss-of-function mechanisms of the GCase enzyme in neurons, thus neglecting the involvement of other cells composing the central nervous system [62]. While the systemic immune dysregulation associated with Gaucher’s disease has been widely investigated [63], only a few papers have postulated the involvement of the observed sustained inflammation in the pathogenesis of Gaucher-associated Parkinson’s disease [12, 64, 65]. Recently, PET scans of GBA mutation carriers revealed microglial activation in Lewy-susceptible brain regions in subjects without either a prodromal or clinical diagnosis of Parkinson’s disease [18], strongly indicating that GCase deficiency can cause microglial dysfunction at the early stages of the disease. Although microglial GCase function has not been dissected at a molecular level, it is well known that when both GBA alleles are mutated, macrophages from Gaucher patients show substantial dysfunction [66], including impairment of autophagy and lysosomal storage and consequent hypersensitivity to proinflammatory stimuli [67]. It is therefore conceivable that similar dysfunction could affect microglia, which share many features with macrophages [68]; if so, the neuroinflammation observed in Gaucher’s disease could be the final manifestation of a loss of homeostasis of the brain-resident macrophages. In line with this view, it has been reported that the expression of *Gba* is higher in microglia than in other cells of the brain [69]. Therefore, partial Gba inhibition could be sufficient to produce functional changes in microglial physiology, substantially affecting brain homeostasis. Indeed, microglia establish a paracrine network of connections with other brain cells mediated by soluble factors [57] and extracellular vesicles [70]) or by direct cell-to-cell contact [56, 71] involving neuronal dendrites or somata; through these connections, specific microglia participate in brain homeostasis and neuronal defense in cases of brain injury or infection [56, 71].

For the first time, we show that under basal conditions, microglia support the NFE2L2-mediated detoxification program in neurons, enriching the microglial arsenal with a new neuroprotective function. Interestingly, this protective function is hampered by GCase inhibition in microglia, with functional consequences on the neuronal redox response and cell survival resulting in increased susceptibility to neurotoxic agents. This mechanism provides a link between neuroprotective microglial function failure and neurodegenerative diseases. The protective mechanism we have discovered relies on direct contact between the two cell lineages mediated by functional actin-dependent structures, namely, the cytoskeleton and filopodia, which are required for the motility of microglial cells [56, 59]. We provide evidence that cell-to-cell contact may be hindered by GCase inhibition, since CBE treatment induced a round morphology in microglial cells (Supplementary Fig. 6) while concurrently reducing cell protrusions, thus curtailing the ability of the microglia to interact with neurons in order to activate the NFE2L2 pathway. The consequence of the reduced NFE2L2 activity is increased susceptibility of neuronal cells to oxidative stress or toxic molecules. Consistent with this conclusion, our coculture experiments demonstrated that reduced GCase activity in microglia is associated with increased sensitivity of dopaminergic neurons to parkinsonian agents such as MPP+ (Fig. 6). Interestingly, the microglial phenotype associated with GCase inhibition seems to be somewhat related to the anti-inflammatory phenotype, since we observed upregulation of anti-inflammatory genes involved in reparative responses (*Arg1* and *Trem2*). This is in line with the phenotype that has been previously described for Gaucher’s cells, the main hallmarks of Gaucher’s disease. Gaucher’s cells are rounded, glucosylceramide-laden dysfunctional macrophages with a distinctive defective phenotype that is similar to an alternative activation state characterized by chronic activation towards the anti-inflammatory phenotype [72, 73]. Accordingly, in genetic murine models of Gaucher’s disease, alveolar and hepatic macrophages show higher expression of genes related to the anti-inflammatory phenotype than their wild-type counterparts [74]. Interestingly, both the lungs and livers of Gba point-mutated mice, which display a mild pathology, exhibit clearly higher expression of *Keap1* (a repressor of Nfe2l2 transcriptional activity) than those of wild-type mice. These data might support the reduction in detoxification pathways shown by our data and the increased susceptibility to toxic molecules observed in GBA models [74].

We speculate that GCase inhibition produces dysfunctional microglia that share pathological features of Gaucher’s cells and consequently fail to activate protective programs in neurons. Therefore, in early neurodegenerative stages, an anti-inflammatory-like microglial phenotype may prevail over the proinflammatory phenotype that has been observed late in the neurodegenerative process, likely as a consequence of the extensive progression of neuronal death [12]. Future studies will clarify the relationship of the CBE-stimulated anti-inflammatory program with the rounded, poorly communicative phenotype of microglia highlighted in our experiments and elucidate its role in neurodegeneration. One possible limitation of the current study is that our observation were made by means of the pharmacological inhibition of the enzyme: future investigations should confirm the microglial phenotype when the GCase activity is reduced by point mutations found in patients; genetic reduction of GCase activity would be important also for understanding the chronic inhibition of the enzymatic activity and the role of our mechanism in the late progression of the disease, eventually assessing the differential effects of GBA mutations in dopaminergic and non-dopaminergic neurons, and their potential role in the behavioral changes observed in the advanced Parkinson’s and Gaucher’s diseases.

The body of evidence generated by our experiments regarding the effects of GCase deficiency in microglia provides a novel mechanistic link between Gaucher variants and Parkinson’s disease. Indeed, our study revealed a novel type of neuroprotection based on direct cell-to-cell contact. We demonstrated that a 50% reduction in microglial GCase activity, which recapitulates the enzymatic activity impairment observed in heterozygotic carriers of severe GBA mutations [75], is sufficient to undermine the neuronal capacity to respond to oxidative stress or neurotoxic insult. Accordingly, in our in vivo experiments, CBE treatment was appropriately titrated [14] to obtain a comparable reduction (Fig. 4). The extent of this impairment was sufficient to significantly affect neuronal survival in vitro in the presence of a neurotoxic stimulus such as MPP+ (Fig. 6). We believe that this vulnerability is especially relevant for dopaminergic neurons, which display high oxidative stress levels and increased susceptibility to neurotoxic stimuli as a consequence of dopamine metabolism [76, 77]. Moreover, the increased oxidative stress together with a reduction in glutathione content in dopaminergic neurons has been associated with compensatory hyperactivation of the NFE2L2 pathway in SNpc neurons [78]. Under these conditions, the slight but constant reduction in NFE2L2 activity induced by GBA mutations in microglia may progressively hinder the full protective response against neurotoxic agents. It is thus conceivable that with reduced NFE2L2 activity, oxidative stress may ultimately have negative consequences on cellular viability, particularly in dopaminergic neurons. Accumulation of damage due to the continuous impairment of NFE2L2 signaling is consistent with the constant low levels of neuronal death occurring during the long process of Parkinson’s pathogenesis, starting years before the onset of clinical manifestations [79]. It is true that not all GBA carriers develop Parkinson’s disease, and we may hypothesize that the increased vulnerability of neurons to neurotoxic stimuli in GBA carriers depends on individual variability in exposure to environmental factors (neurotoxic stimuli) and/or on different levels of oxidative stress associated with genetic factors. Both conditions may account for the different penetrance of specific GBA mutations in the development of the neurodegenerative phenotype.

## Conclusions

**Fig. 9:**
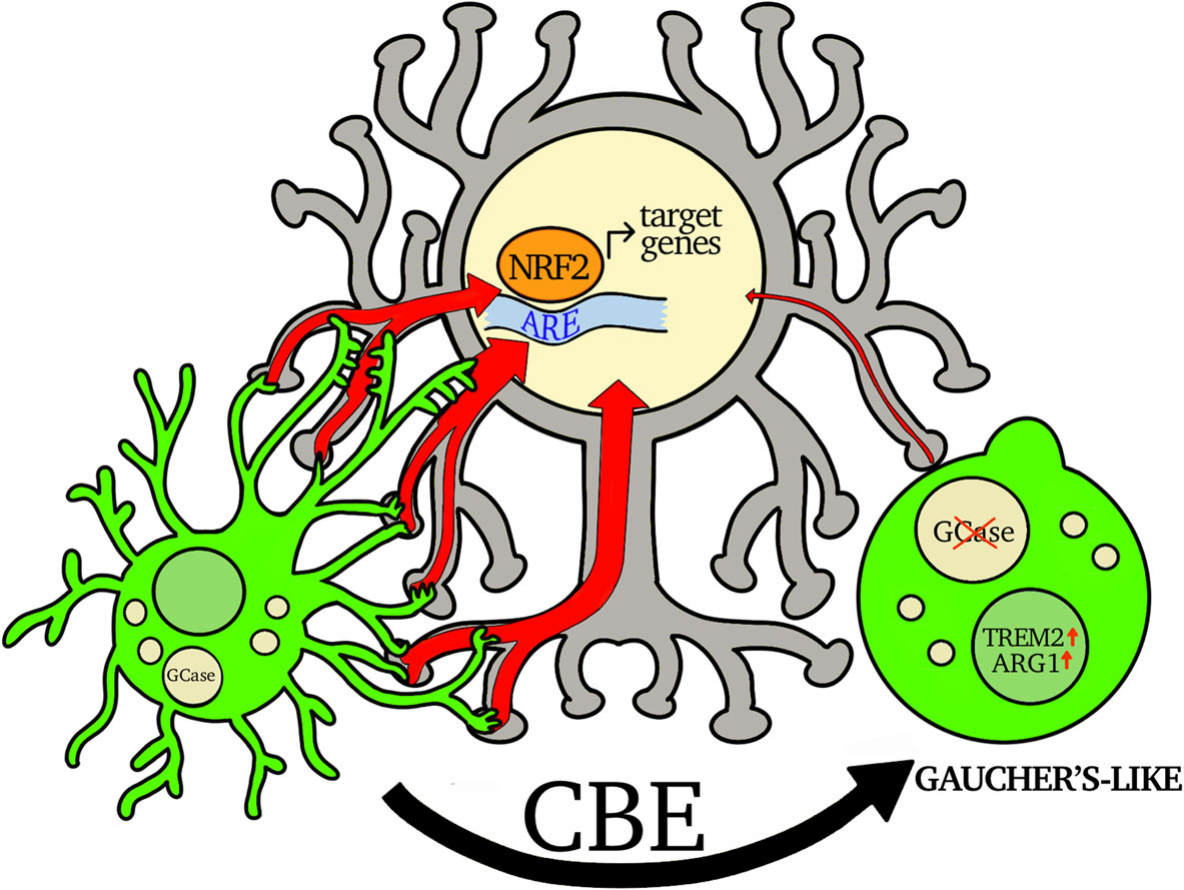
Schematic representation of GCase inhibitions on microglia and neuron communication. Microglia, through actin-dependent structures, directly contact neurons and induce a detoxification response by increasing the NFE2L2 signaling pathway. Inhibition of GCase activity by CBE treatment produces a morpho-functional change in microglia which acquire a morphology and gene expression pattern reminiscent of Gaucher’s cells. This phenotype shift hampers the neuroprotective microglia-neuron communication thus inducing a phenotype in dopaminergic neurons characterized by increased susceptibility to oxidative stress or toxic insults

In conclusion, numerous studies have highlighted the neuronal effects of GBA mutations on lysosomal and mitochondrial neuronal functions that lead to increased α-synuclein deposition and vulnerability to neurotoxic insults. In our study, we demonstrated that GCase inhibition impairs the ability of microglia to induce a detoxification response through modulation of the NFE2L2 signaling pathway in neurons (Fig. 9). Alterations in this protective mechanism may increase the risk of neurodegeneration induced by toxic molecules and oxidative stress, which is especially important for dopaminergic neurons characterized by elevated oxidative metabolism. Unveiling this mechanism might contribute to identification of novel targets to restore microglial neuroprotective functions and prevent the onset of neurodegeneration in Gaucher’s and Parkinson’s diseases.

## Supplementary Information

**Additional file 1: Supplemental Figure 1.** SK-ARE-*luc2* clone selection. Following transfection with the pARE-*luc2*-ires-tdTomato plasmid, SK-N-BE cells were subject for 4 weeks to a selective pressure with G418 and two representative clones #1.2 and #3 tested for the ability to report NFE2L2 upregulation 2, 6 or 24 hours after the treatment with 80 μM tBHQ or vehicle. Clone #1.2 was selected, amplified and used in the present study. Luciferase enzyme activity expressed as relative luciferase units (RLU) per μg protein, data are mean values ± SEM (n = 3) of a single experiment, which is representative of at least two other independent experiments. *p<0.05 vs vehicle calculated by one-way ANOVA followed by Tukey’s multiple comparison test. **Supplemental Figure 2.** GCase inhibition in mice and SK-ARE-*luc2* cells treated with CBE. (**A**) Residual activity of GCase in the brain of mice treated with 100 mg/kg CBE for three days. (**B**) Residual activity of GCase in SK-ARE-*luc2* cells treated with 200 μM CBE for 48 hours. Data are mean values of the enzymatic activity quantified as μmol 4-MU generated in 1-hour reaction per μg of proteins expressed as % of the activity detected versus vehicle treated animals (A) or cells (B) ±SD of n=2 in duplicate (*in vivo* experiments), n=2 in triplicate (cell culture experiments). **p < 0.01, ***p<0.001 *versus* vehicle calculated by unpaired t-test. **Supplemental Figure 3.** Different ratio of BV-2:SK-ARE-*luc2* cocultures differentially modulate neuronal NFE2L2 activity. Luciferase activity is expressed as RLU is reported on the graph as FC on monoculture; bars are mean values ±SD of n=5 measures in triplicate. ***p<0.001 by one-way ANOVA followed by Dunnet’s multiple comparisons test. **Supplemental Figure 4.** Nuclear localization of NFE2L2 in neuronal-microglia culture treated with CBE. Representative immunocytochemistry analysis of SK-N-BE and BV-2 cell lines in monoculture and coculture, treated with 200 μM CBE or vehicle for 48 hours; cells were co-stained with anti- NFE2L2 (red) and anti-CD11b antibodies (green), and with DAPI (blue). **Supplemental Figure 5.** GCase inhibition dampen neuroprotective functions of microglia. Representative single parameter histograms of flow cytometry analyses related to propidium iodide fluorescence: (A) SK-N-BE treated with 0.5 mM MPP+ or vehicle for 24 hours; (B) SK-N-BE cultured alone or in coculture with BV-2 and treated with 0.5 mM MPP+ for 24 hours; (C) cocultures treated with 200 μM CBE for 48 hours or (D) with 200 μM CBE for 48 hours and 0.5 mM MPP+ for 24 hours. **Supplemental Figure 6.** GCase inhibitions in primary microglia. Representative images of primary microglia marked with GFP showing that the treatment with 200 μM CBE for 48 hours increases a microglia sub-population characterized by a round shaped morphology.

## Data Availability

The data that support the findings of this study are available from the senior author (paolo.ciana@unimi.it) upon reasonable request.

## Abbreviations

BLI: Bioluminescence imaging
CBE: Conduritol-B-epoxide
CCD: Charge-coupled device
FC: Fold change
FBS: Fetal bovine serum
GCase: β-Glucocerebrosidase
i.p.: Intraperitoneal
MPP+: 1-Methyl-4-phenylpyridinium
MPTP: 1-Methyl-4-phenyl-1,2,3,6-tetrahydropyridine
NFE2L2: Nuclear factor erythroid 2-related factor 2
PET: Positron emission tomography
ROI: Regions of interest
RT: Room temperature
RT-PCR: Real-time polymerase chain reaction
SNpc: Substantia nigra pars compacta
tBHQ: Tert-butylhydroquinone
Th: Tyrosine hydroxylase
μglia: Microglia
