# Supplementary material for: Inhibition of microglial β-glucocerebrosidase hampers the microglia-mediated antioxidant and protective response in neurons
Source: J Neuroinflammation. 2021 Sep 22;18:220. doi: 10.1186/s12974-021-02272-2 (PMC8459568; doi:10.1186/s12974-021-02272-2)
Supplement: Supplementary file 1 — Additional file 1: Supplemental Figure 1. SK-ARE-luc2 clone selection. Following transfection with the pARE-luc2-ires-tdTomato plasmid, SK-N-BE cells were subject for 4 weeks to a selective pressure with G418 and two representative clones #1.2 and #3 tested for the ability to report NFE2L2 upregulation 2, 6 or 24 hours after the treatment with 80 μM tBHQ or vehicle. Clone #1.2 was selected, amplified and used in the present study. Luciferase enzyme activity expressed as relative luciferase units (RLU) per μg protein, data are mean values ± SEM (n = 3) of a single experiment, which is representative of at least two other independent experiments. *p<0.05 vs vehicle calculated by one-way ANOVA followed by Tukey’s multiple comparison test. Supplemental Figure 2. GCase inhibition in mice and SK-ARE-luc2 cells treated with CBE. (A) Residual activity of GCase in the brain of mice treated with 100 mg/kg CBE for three days. (B) Residual activity of GCase in SK-ARE-luc2 cells treated with 200 μM CBE for 48 hours. Data are mean values of the enzymatic activity quantified as μmol 4-MU generated in 1-hour reaction per μg of proteins expressed as % of the activity detected versus vehicle treated animals (A) or cells (B) ±SD of n=2 in duplicate (in vivo experiments), n=2 in triplicate (cell culture experiments). **p < 0.01, ***p<0.001 versus vehicle calculated by unpaired t-test. Supplemental Figure 3. Different ratio of BV-2:SK-ARE-luc2 cocultures differentially modulate neuronal NFE2L2 activity. Luciferase activity is expressed as RLU is reported on the graph as FC on monoculture; bars are mean values ±SD of n=5 measures in triplicate. ***p<0.001 by one-way ANOVA followed by Dunnet’s multiple comparisons test. Supplemental Figure 4. Nuclear localization of NFE2L2 in neuronal-microglia culture treated with CBE. Representative immunocytochemistry analysis of SK-N-BE and BV-2 cell lines in monoculture and coculture, treated with 200 μM CBE or vehicle for 48 hours; cells were co-sta [file 12974_2021_2272_MOESM1_ESM.docx]

**Supplementary figures**


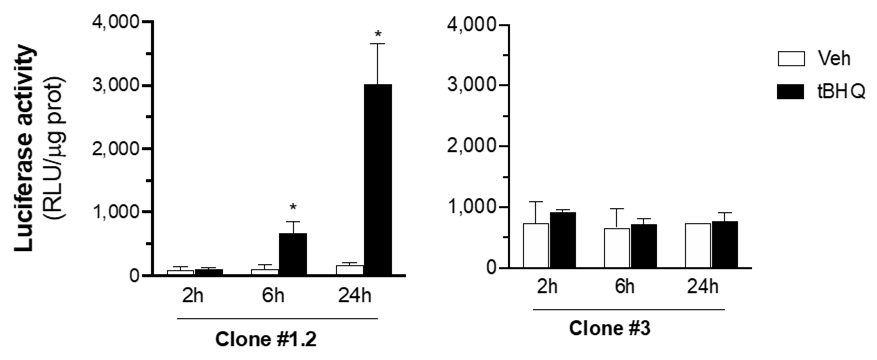


**Supplementary Figure 1. SK-ARE-*luc2* clone selection.** Following transfection with the pARE-*luc2*-ires-tdTomato plasmid, SK-N-BE cells were subject for 4 weeks to a selective pressure with G418 and two representative clones #1.2 and #3  tested for the ability to report NFE2L2 upregulation 2, 6 or 24 hours after the treatment with 80 µM tBHQ or vehicle. Clone #1.2 was selected, amplified and used in the present study. Luciferase enzyme activity expressed as relative luciferase units (RLU) per μg protein, data are mean values ± SEM (n = 3) of a single experiment, which is representative of at least two other independent experiments.  **p*<0.05 vs vehicle calculated by one-way ANOVA followed by Tukey’s multiple comparison test.


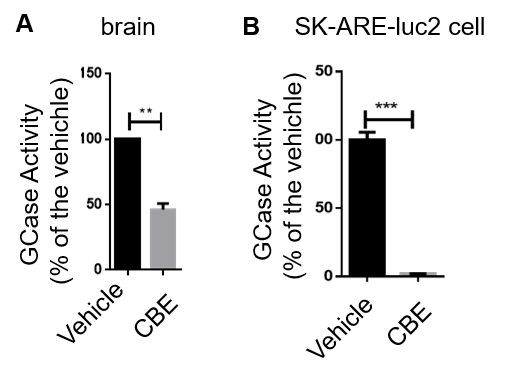


**Supplementary Figure 2.** **GCase inhibition in mice and SK-ARE-*luc2* cells treated with CBE.**

(**A**) Residual activity of GCase in the brain of mice treated with 100 mg/kg CBE for three days. (**B**) Residual activity of GCase in SK-ARE-*luc2* cells treated with 200 µM CBE for 48 hours. Data are mean values of the enzymatic activity quantified as µmol 4-MU generated in 1-hour reaction per µg of proteins expressed as % of the activity detected versus vehicle treated animals (A) or cells (B) ±SD of n=2 in duplicate (*in vivo* experiments), n=2 in triplicate (cell culture experiments). ***p* < 0.01, ****p*<0.001 *versus* vehicle calculated by unpaired t-test.

**
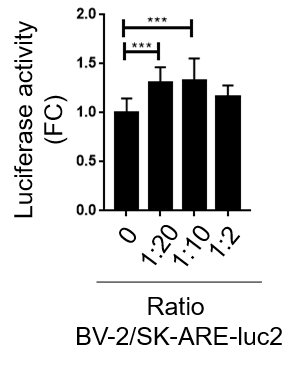
**

**Supplementary Figure 3. Different ratio of BV-2:SK-ARE-*luc2* co-cultures differentially modulate neuronal NFE2L2 activity.** Luciferase activity is expressed as RLU is reported on the graph as FC on monoculture; bars are mean values ±SD of n=5 measures in triplicate. ****p*<0.001 by one-way ANOVA followed by Dunnet’s multiple comparisons test.

**
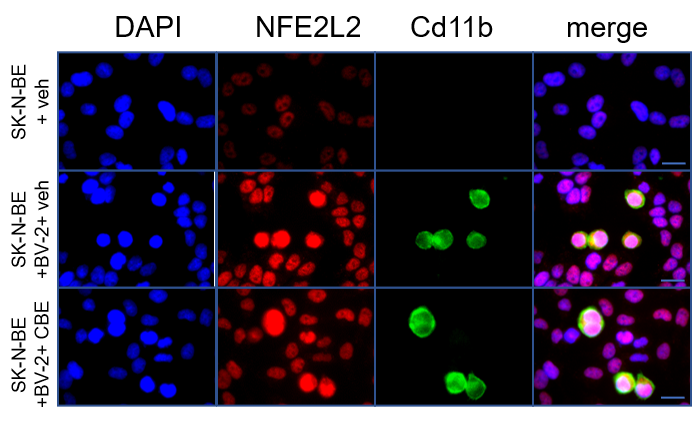
**

**Supplementary Figure 4: Nuclear localization of NFE2L2 in neuronal-microglia culture treated with CBE.** Representative immunocytochemistry analysis of SK-N-BE and BV-2 cell lines in monoculture and co-culture, treated with 200 µM CBE or vehicle for 48 hours; cells were co-stained with anti- NFE2L2 (red) and anti-CD11b antibodies (green), and with DAPI (blue).


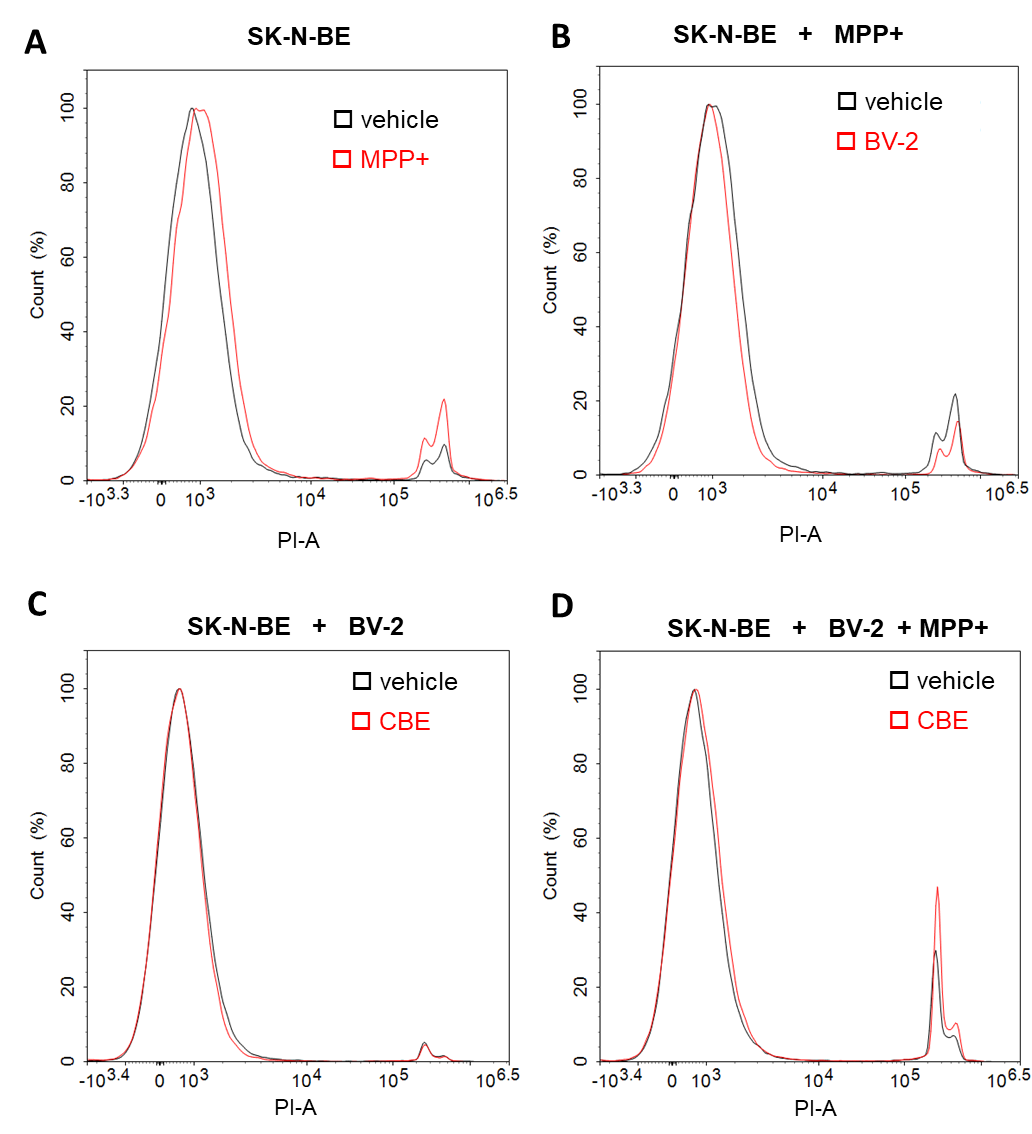


**Supplementary Figure 5: GCase inhibition dampen neuroprotective functions of microglia**. Representative single parameter histograms of flow cytometry analyses related to propidium iodide fluorescence: (A) SK-N-BE treated with 0.5 mM MPP+ or vehicle for 24 hours; (B) SK-N-BE cultured alone or in co-culture with BV-2 and treated with 0.5 mM MPP+ for 24 hours; (C) co-cultures treated with 200 µM CBE for 48 hours or (D) with 200 µM CBE for 48 hours and 0.5 mM MPP+ for 24 hours.


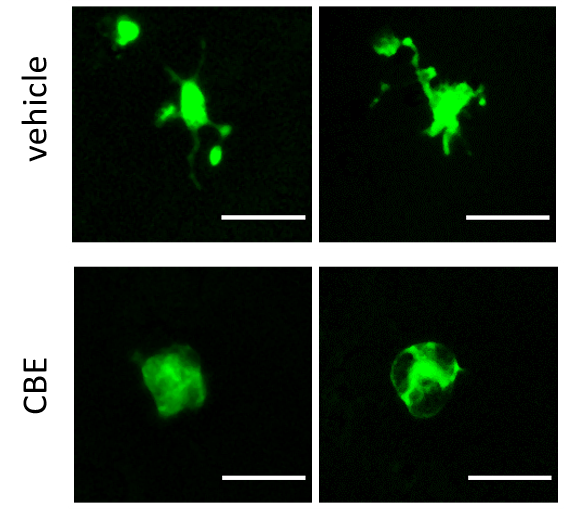


**Supplementary Figure 6: GCase inhibitions in primary microglia**. Representative images of primary microglia marked with GFP showing that the treatment with 200 µM CBE for 48 hours increases a microglia sub-population characterized by a round shaped morphology.
